# Predator-Prey in Tumor-Immune Interactions: A Wrong Model or Just an Incomplete One?

**DOI:** 10.3389/fimmu.2021.668221

**Published:** 2021-08-31

**Authors:** Irina Kareva, Kimberly A. Luddy, Cliona O’Farrelly, Robert A. Gatenby, Joel S. Brown

**Affiliations:** ^1^EMD Serono, Merck KGaA, Billerica, MA, United States; ^2^Department of Cancer Physiology, H. Lee Moffitt Cancer Center, Tampa, FL, United States; ^3^School of Biochemistry and Immunology, Trinity College Dublin, Dublin, Ireland; ^4^Department of Integrated Mathematical Oncology, Moffitt Cancer Center, Tampa, FL, United States

**Keywords:** immunoediting, first principles, cancer ecology, tumor-immune interactions, predator-prey dynamics, immune-web

## Abstract

Tumor-immune interactions are often framed as predator-prey. This imperfect analogy describes how immune cells (the predators) hunt and kill immunogenic tumor cells (the prey). It allows for evaluation of tumor cell populations that change over time during immunoediting and it also considers how the immune system changes in response to these alterations. However, two aspects of predator-prey type models are not typically observed in immuno-oncology. The first concerns the conversion of prey killed into predator biomass. In standard predator-prey models, the predator relies on the prey for nutrients, while in the tumor microenvironment the predator and prey compete for resources (e.g. glucose). The second concerns oscillatory dynamics. Standard predator-prey models can show a perpetual cycling in both prey and predator population sizes, while in oncology we see increases in tumor volume and decreases in infiltrating immune cell populations. Here we discuss the applicability of predator-prey models in the context of cancer immunology and evaluate possible causes for discrepancies. Key processes include “safety in numbers”, resource availability, time delays, interference competition, and immunoediting. Finally, we propose a way forward to reconcile differences between model predictions and empirical observations. The immune system is not just predator-prey. Like natural food webs, the immune-tumor community of cell types forms an immune-web of different and identifiable interactions.

## Introduction

Evolution is the change in a population’s heritable traits over time subject to selection pressures through population turnover. Co-evolution occurs when close interactions between two or more species affect each other’s selective pressures. In the co-evolutionary arms race between a pathogen and a host, pathogens often replicate faster, and therefore can evolve and adapt rapidly, while a host cannot. For instance, the invasive ash borer beetle has decimated its host, the ash trees of North America. Once infected, mortality rates are near 100% (1). In time, the ash trees may evolve resistance, but this will require decades as new trees grow from seedlings to mature trees. In contrast, even though single-celled pathogens can evolve rapidly relative to their hosts, vertebrate immune systems break a conundrum of ecological and evolutionary time scales.

Vertebrates employ specialized lymphocytes (B cells and T cells) equipped with diverse recognition receptors (BCR and TCR, respectively). These receptors are unique for each B or T cell clone and are generated stochastically early in the cell’s development (1, 2). Populations of immune cells will change and respond when a pathogen evolves, or when a novel pathogen arises. The co-evolutionary arms race therefore occurs within the human body between immune cells and evolving pathogens or transformed cells. The intrinsically dynamic nature of the immune system makes it an excellent tool for the host to counter cancer cells. But understanding the circumstances under which the immune system succeeds or fails to contain a cancer population remains challenging.

Mathematical modeling has been and continues to be employed to understand the underlying dynamics, to help formalize hypotheses, and to evaluate and improve treatment options (3–5). This is critically important in immuno-oncology, where clinicians must consider the tumor’s response to therapy as well as the immune system’s (4, 6, 7). Insights from ecological literature provide new hypotheses for managing cancer as well (8–12). A rich body of mathematical modeling of species interactions can be found within population ecology (4). Examples of ecological interactions include competition, mutualisms, and predator-prey interactions. Such models can be constructed to reveal the key mechanisms underlying the dynamics of different cell types within the human body. For example, the interactions between the community of immune cells and pathogens or cancer cells resembles a predator-prey system. As “prey” for the immune system, cancer cells differ from normal cells due to accumulated mutations and changes in antigen levels that can activate predatory cytotoxic T lymphocytes (CTL) (13–15). As the “predator”, tumor-specific CTLs’ proliferation and survival rates depend on the presence and interactions with their prey.

The predator-prey analogy is an attractive framework that has been modeled extensively in ecology, offering a strong foundation for oncology modelers (16). However, it is an imperfect comparison. Several key assumptions and predictions of predator-prey models do not apply to tumor-immune biology, and these differences highlight unique avenues for increasing the effectiveness of cancer immunotherapy. In the following, we explore parallels between classic predator-prey systems and the immune-cancer interactions and compare these models to known biology and experiments of immuno-cancer dynamics. We then explore interactions between tumor cells and T cells as both predator-prey systems and as competitive systems embedded within the larger community of immune cells. We frame these interactions in light of recent work highlighting the significant impact of immunometabolism on immune cell function. Cancer immunology has reached an inflection point, where prior views of treatment resistance, which are based largely on other treatment modalities (e.g. chemotherapies and targeted therapies), need to be expanded. Currently, resistance mechanisms are seen as novel therapeutic targets with little understanding for how they arise or what may come next. Here we begin to address this by providing a more complete view of tumor immune dynamics that is important for mathematical modelers, for researchers, and for clinicians seeking to improve the efficacy of cancer immunotherapy. We conclude with a discussion of avenues for future modelling, research, and clinical applications.

## Predator-Prey Dynamics in Nature

### Lotka-Volterra Predator-Prey Model

In the 1920s, Alfred Lotka and Vito Volterra independently developed the first predator-prey model. Lotka aimed to describe population dynamics between plants and herbivorous animals. Volterra was investigating the unexpected oscillatory dynamics of predatory fish in the Adriatic sea that was observed throughout the 1910s despite the suspension of fishing during World War I (17). The original model had four terms as follows:

$$\begin{array}{l} x΄=ax-bxy\\ y΄=-cy+d(bxy), \end{array}$$

where *x* is the population size of prey and *y* is the population size of the predator, *a* is the per capita birth rate of the prey, *b* is the encounter probability between predator and prey (*bx* is the rate at which a predator individual kills prey), *c* is the per capita death rate of the predator, and *d* is the conversion efficiency of prey consumed by a predator into new predators.

The Lotka-Volterra (L-V) predator-prey model assumes that 1) the prey population grows exponentially in the absence of the predator, 2) each predator individual kills a fixed proportion of the prey population per unit time, 3) a predator’s birth rate increases linearly with the rate of prey consumption, and 4) predators have a constant death rate. This model has a neutrally stable equilibrium of prey and predator population sizes. This means that for any initial starting point, the prey and predator population sizes oscillate perpetually. The magnitude of this oscillation increases with the distance of initial conditions from the equilibrium point x* = *c/d*, y* = *a/b*.

The L-V predator-prey model quantitatively frames two important concepts common to all predator-prey models: the functional response and the numerical response.

The functional response is the rate at which an individual predator (e.g., a cytotoxic cell) kills prey (e.g., cancer cells) as a function of prey density (e.g., cancer cells per unit volume). When multiplied by the number of predators, the functional response determines the prey’s death rate. This relates to the ratio of effector to target cells measured in immunology. Cytotoxic immune cells kill at a rate influenced by the density of target cells. In the L-V model, the predator’s functional response is *bx*.

The numerical response links the functional response to the predator’s growth rate (18). Generally, the growth rate of the predator population increases with the rate of prey consumption. The more you eat the more you grow, a concept that, in predator-prey modeling, has also been described as the biomass conversion of prey into predators (19). In this case, the conversion of prey consumed (the functional response) into predator births is *dbx*.

**Figure 1:**
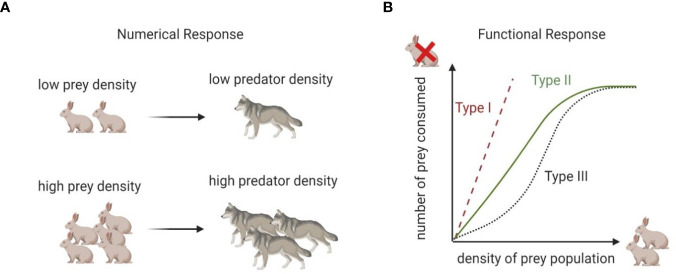
Numerical vs functional response. **(A)** Numerical response describes conversion of prey density into predator density. **(B)** Functional response captures relationship between rate of consumption and food density. Type 1 response implies that rate of consumption of predator is proportional to prey density. Type II response implies that the number of prey consumed increases rapidly with increased prey population density but plateaus at a carrying capacity. Type III response is similar to Type II but assumes that at low prey density rate of prey consumption is slower than in Type II.

Together, the functional and numerical responses define classic predator-prey interactions, in which predators kill and consume their prey. A schematic description of differences between functional and numerical responses is shown in **Figure 1**; a more detailed description of different types of functional responses will be given in the next section. Despite its simplicity, the L-V model provided a mechanistic explanation for the oscillatory behavior of predatory fish (initially observed by Volterra’s son-in-law, marine biologist Umberto D’Ancona). It revealed how the four parameters included in the model were sufficient to qualitatively reproduce observations without additional assumptions regarding other properties of the environment.

### General Predator-Prey Model

Mathematically, a more general form for the functional response can be:

$$f(x)=\frac{bx^{\alpha }}{1+bhx^{\alpha }},$$

where *b* is a predator’s encounter probability on prey (same as in the L-V model), and α scales the predator’s encounter rate (*bx* ^α^) by allowing for an attraction effect (α>1) or a dilution effect (α<1). An attraction effect (which can be driven by an immune cell chemo-attractant) increases the probability that an immune cell will encounter a target cell. A dilution effect decreases the probability that an immune cell will encounter a particular tumor cell. *h* is the handling time that a predator takes to capture and consume an encountered prey, which can be interpreted as the time an immune cell spends at the immunological synapse.

The functional response can take on a number of forms: Type I: (*h*=0 and α=1), where no time is spent at the immunological synapse and no attractive forces or dilution effects occur; Type II: (*h*>0, 0<α≤1), or saturating response, where immune cells spend some time at the immunological synapse and there is a dilution effect, and Type III: (*h*>0, α>1), the sigmoidal response, where immune cells spend time at the immunological synapse and there is a chemo-attractant. Each form has different properties (20) summarized in **Figure 1**. The classification by types was proposed by C.S. Holling (21), and thus these functional responses are frequently referred to as Holling Type responses.

These variants of the functional response better encompass the range of empirical observations in natural predator-prey systems, as each implies different underlying mechanisms for prey detection and capture by the predators. For instance, prey pursued by a predator with a Type I functional response experience neither safety nor danger in numbers; the rate of prey consumption is proportional to prey density. When the predator has a Type II functional response, the prey experience safety in numbers through the dilution effect. The likelihood of a given prey individual experiencing predation declines with prey density. This tends to destabilize the population dynamics of prey and predator, leading to oscillations rather than stable coexistence. The Type III functional response is often described as the “controlling functional response” because, at low densities, the prey experience danger in numbers, and the predators can act to strongly suppress the prey, maintaining them at low densities. At higher prey densities, this control is lost, and the prey now revert to experiencing safety in numbers. With a type III functional response, a predator individual’s consumption rate of prey at first accelerates and then decelerates with increasing prey density (**Figure 1**).

With these possible forms for the functional response, an expanded model can include diverse features, such as the following (22):

$$\begin{array}{c} \underset{prey}{\underset{︸}{x'}}=\underset{growthwithAlleeandcarryingcapacity}{\underset{︸}{ax(x-L)(K-x)}}-\underset{deathbypredation}{\underset{︸}{f(x)y}}\\ \underset{prey}{\underset{︸}{y'}}=\underset{conversionofpreybiomassintopredator}{\underset{︸}{(d-g)f(x)y}}-\underset{naturaldeath}{\underset{︸}{cy}} \end{array}$$

where *x* is the population of the prey, *y* is the population of the predator, *a* is the prey growth rate, *K* is the carrying capacity of the population of prey, and *L* is the prey’s extinction threshold due to the Allee effect (22, 23), which will be discussed below. The prey is consumed by each predator individual at a rate of *f*(*x*) and is converted into predator biomass at rate *d*; the predator dies at rate *c*, and there is a probability *g* of lethal injury to the predator when capturing prey.

The model includes a carrying capacity with limits to growth (*K*-*x*), and an Allee effect represented by (*x*-*L*). With an Allee effect, there can exist an extinction threshold for the prey’s population size, below which the prey becomes extinct and above which the population grows to a carrying capacity in the absence of predators (24). In the predator equation we include the risk of injury to capturing prey. Notably, if *g* > *d*, then the predator has negative fitness from capturing prey and the predator should avoid such prey. More generally we expect *d* >> *g*. However, *g* > *d* may apply at times to immune cells, such as T-cells attacking cancer cells and as a result, experiencing injury or exhaustion. For instance, tumor cell expression of programed death protein-1 (PD-1) actively inhibits T cell function following ligation of the programmed death receptor.

The more general predator-prey model can assess the consequences of different assumptions. Parameter values determine the predator-prey population dynamics, the presence of equilibrium points, and the stability of these equilibria. When applied to immune-cancer interactions, the underlying mechanisms behind each parameter can be targeted clinically and evaluated based on how a therapy changes predicted output.

**Figure 2:**
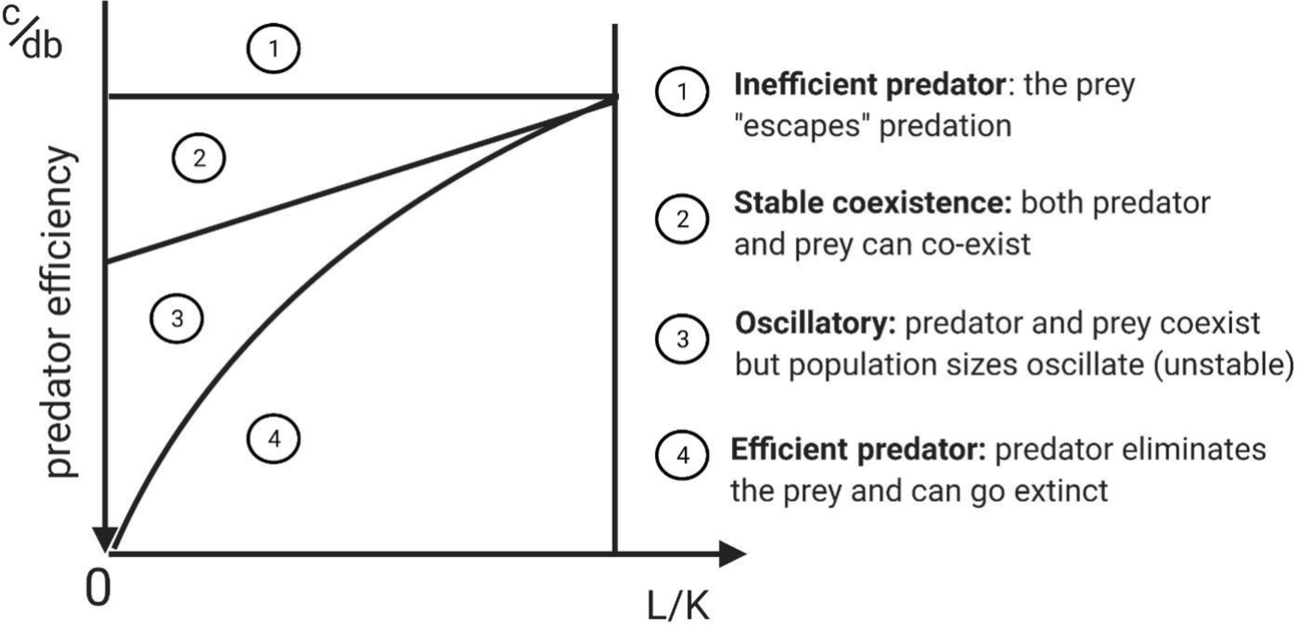
Complete phase-parameter portrait of Lotka-Volterra model with Allee effect that captures the possible dynamical regimes possible in the model subject to variation of predator inefficiency (c/db) and the ratio of the extinction threshold to carrying capacity (L/K). In this figure the predators have a type I functional response and so there is no handling time (h=0). Adapted from original study by (23), reprinted in (24), Section 3.5.5. The diagram highlights that there is a predictable sequence of regimes between predator efficiency and inefficiency.

Depending on parameter values, the above model results in five types of dynamical behaviors that result in four qualitatively different outcomes. All have been observed in natural predator-prey systems. The specific outcomes depend on *predator efficiency*, which can be defined by the predator’s benefit to cost ratio [(*bd)/c*] where *bd* is the fitness reward from encountering and capturing prey, and *c* is the fitness cost. The ratio of the extinction threshold to the carrying capacity, *L/K* (*L* < *K*), is a second factor. When coupled with predator efficiency, these determine the outcome of the predator prey interaction (**Figure 2** shows how various combinations of predator efficiency and *L/K* determine dynamical regimes).

If the predator is very efficient (high value for *bd*), it eliminates the prey (**Figure 2**, region 4). As the value of *bd* decreases and the predator becomes less efficient, an oscillatory regime appears, as the prey and predator populations “chase” each other in a manner reminiscent of the original L-V predator-prey model (**Figure 2**, region 3). As predator efficiency decreases further, the prey and the predator coexist at a stable equilibrium (**Figure 2**, region 2). Finally, as predator efficiency decreases further, it cannot be supported by the prey. The predators go extinct and the prey population can “escape” (**Figure 2**, region 1). This sequence of regimes resulting from diminishing predator efficiency happens to describe the phases of immunoediting, suggesting a parallel between predator-prey and cancer-immune interactions.

## Immune System as Predator and Cancer Cells as Prey

The relationship between cytotoxic immune cells and tumor cells seems to resemble a predator prey interaction (25–28). Once activated, immune cells (the predators) search for cells expressing their cognate antigen (the prey). After the target has been identified, immune cells physically bind with and kill the target cell. Furthermore, the dynamics of T cell populations are often dependent on the presence of the pathogen. For example, antigen detection is followed by rapid clonal expansion of relevant T cell populations. As pathogens are cleared, antigen load diminishes, and T cell populations contract (29). A summary of similarities and differences between cancer-immune and prey-predator interactions is given in **Table 1**.

**Table 1:** Key differences between the assumptions underlying classic predator-prey systems, and corresponding mechanisms in tumor-immune interactions where tumor cells are prey and immune cells are the predators.

Biological mechanism	Prey-predator	Tumor-immune
**Prey growth**	• does not depend on predator • limited by a carrying capacity • depends on nutrient availability	• independent of cytotoxic lymphocyte activation • limited by a carrying capacity • depends on nutrient availability (8)
**Prey death**	• natural death • death by the predator	• natural death • death by the predator (immune) • once a T cell is activated, kill rate is proportional to probability of encountering a cancer cell (9) • there is “handling time” for cancer cell kill, creating safety in numbers for cancer cells (10)
**Predator growth**	• predator works “on commission”: proliferation depends on efficiency of predation (conversion of prey biomass into predator biomass) • there is no distinction between proliferation and activation	• immune cells do not work on commission: killing a cancer cell does not directly increase likelihood of T cell proliferation (2) • there is a distinction between proliferation and activation (2) • killing cancer cells can create a “vaccinating effect” through increasing proliferation of antigen presenting cells (APCs) that can increase T cell population, i.e., indirect increase in T cell population size (11) • predator growth and activation may be impaired by low nutrient availability (12)
**Predator death**	• Predator does not have a limit on prey kill • Predator dies in the absence of prey	• T cell can kill a limited number of cancer cells (9) • Activated T cells die in the absence of target cells (13) • T cells can become tolerized (14)

The basic assumptions of a classic predator-prey model (with a few caveats) fit our conceptual understanding of tumor immune dynamics. The underlying model assumptions can be reworded as follows: 1) tumor cell populations grow near exponentially in the absence of the immune system (at least up to some carrying capacity, with the possibility of an extinction threshold), 2) each immune cell kills a fixed proportion of the tumor population per unit time, 3) the immune cell’s birth rate increases with the rate of killing cancer cells, and 4) the immune cells have a constant death rate. However, assumption 1 ignores the possibility that cancer cell proliferation rates may show a hyper-proliferative response to the introduction of immune cells. This has been observed in c. 7% of patients receiving immunotherapy (30). Assumption 2 ignores the diversity of immune cells. Assumption 3 implies immune cells gain energy from killing target cells (more on this below). And assumption 4 ignores tolerance, loss of function through various mechanisms of reversible and irreversible immune cell exhaustion, and memory formation. All of these do not necessarily disqualify the applicability of the predator-prey framework to cancer-immune interactions, but they do require further elaboration.

Like in other predator-prey systems, “immune efficiency” is a critical contributor to the outcomes of the immune-cancer cell interaction. Factors that affect immune efficiency include, but are not limited to, genetic and phenotypic variability of cancer cells (31–33), competition for shared nutrients (34), altered antigenicity, increased regulatory immunity that is frequently observed in tumor microenvironments, impact of acidic microenvironment on immune cell function (35–37), as well as factors that may augment phenotypic plasticity of cancer cells in the presence of the immune system, or “ecology of fear”.

### Genetic Factors

Cancer cells are genotypically and phenotypically diverse, leading to variations in the ability of cytotoxic lymphocytes to recognize and kill them. Several studies have suggested that tumors with high mutational burdens are more likely to be recognized and eliminated by the immune system and are thus more responsive to immunotherapy (38–40). Within the framework of immunoediting, cytotoxic lymphocytes eliminate cancer cells with recognizable epitopes, leaving behind cancer cells to which the immune system is blind, thereby “editing” the tumor and allowing it to progress (41). Additionally, it has been shown that immunoediting selects for cancer cells that suppress the immune response through various mechanisms (PDL1, IDO, sMICA, HLA-E, suppressive cytokines, etc.), as well as resistance to apoptosis (42, 43). The immune system’s functional and numerical responses to cancer cells become paramount.

### Competition for Resources

Cancer cells increase glycolysis, which reduces glucose concentration in the tumor microenvironment. For T cells to activate and proliferate, they too must take up glucose and initiate glycolysis. Theoretic (44) and experimental (34) research has shown that glucose deprivation and metabolite accumulation reduce T cell functioning. Nutrient deprivation compromises the immune cells’ ability to move, kill, and even differentiate (45–48). Cancer cells consume other resources needed by immune cells including oxygen, tryptophan, glutamate, folic acid and various amino acids, among others. Ecologically, cancer cells are not just prey for immune cells, they are also competitors.

### Increase in Immune Regulation

The whole organism maintains a homeostatic balance between cytotoxic and regulatory immunity, which is critical for maintaining adequate immune response to fight disease, such as cancer, and to prevent immuno-pathologies. Key players include regulatory T cells (Tregs), myeloid derived suppressor cells (MDSC), polymorphonuclear neutrophils (PMN), and subsets of NK and B cells (49–53). Tregs regulate cytotoxic immunity by suppressing effector cell survival and function, and by directly killing effector T cells *via* granzyme and perforin-dependent mechanisms (54–56). Within the context of a food chain in nature, one can view regulatory immunity as “the predator’s predator”. It is possible that, as the population of cytotoxic immune cells becomes too proliferative, oscillatory predator-prey dynamics may develop between these T cells and cancer cells *via* exaggerated numerical responses. The whole immune system may shackle the efficacy of cytotoxic immune cells to prevent oscillatory dynamics, auto-immunity, and other ill effects to the whole organism that could emerge if the predatory immune cells were too efficient and too proliferative. In nature, predators may have their own predators, but the emerging food-webs of predation, competition and mutualisms among species has not been honed for some greater homeostatic need of the whole ecological community, unlike the community of immune cells within the ecosystem of the body.

### Ecology of Fear

The ecology of fear considers the non-lethal effects that predators have on their prey. Such effects generally involve prey using vigilance or habitat selection to reduce their likelihood of suffering predation (57–59). For instance, fear of large carnivores can lead to changes in feeding patterns of smaller carnivores, such as raccoons, which in turn may affect the behavior of their prey (60). The ecology of fear will manifest in tumors if cancer cells respond behaviorally or phenotypically to the presence of cytotoxic immune cells. For instance, cancer cell motility increases in immune-conditioned media (61–65). The epithelial-to-mesenchymal transition (EMT) of cancer cells can compromise immuno-surveillance and the ability of immune cells to attack cancer cells (66, 67). Increased glycolysis by cancer cells in response to immune cells may decrease pH and create an acidic moat around the cells that inhibits the action of T cells (37, 68, 69). Such “vigilance behavior” can be manifested as immune cells introduce interferons or interleukins into the environment, while cancer cells deploy cytokine receptors to detect these to increase awareness of the presence of predatory immune cells.

### Biomass Conversion of Prey Into Predators Works Differently in the Immune System

There are three properties of the immune-cancer interaction that make it unlike predator-prey dynamics in nature. Each property relates to how the immune system determines the per capita growth rates of cytotoxic immune cells.

First, classic predator-prey type models assume a direct conversion of consumed prey biomass into that of the predator. The killing of prey directly influences the predator’s expected per capita growth rate, either by increasing birth rates or decreasing death rates. Predators in most ecological systems work on “commission”, whereby they and their prey are linked *via* the functional response, which is the predator’s fitness reward, and the prey’s fitness penalty. This is not so for cytotoxic immune cells. Killing of cancer cells requires expenditure of resources with no direct compensatory nutrient gain from the dead cell. Thus, the growth rate of cytotoxic immune cells is not directly influenced by their kill rate of cancer cells. This is highlighted in sterile immune responses, where immune cell numbers increase despite the lack of pathogens, and during chronic infections, when immune cell numbers decrease even when the pathogen has not been cleared (70).

Instead, the growth of populations of these T cells is determined largely by immunogenicity of dominant epitopes, strength of antigenic discontinuity, duration and rate of antigen increases, and external signaling (71–74). The immunogenicity and presence of the dominant epitopes can stimulate the proliferation of cytotoxic cells, and while the presence of these epitopes might increase with the killing of cancer cells, it is more tied to the presence rather than the death rate of cancer cells. Antigenic discontinuity recognizes that immune cell proliferation can increase (or decrease) with abrupt changes in the presence of antigens. Under the Growth Threshold Conjecture (71), the functional and numerical response of T cell populations will be elastic: if a pathogen proliferates slowly, it induces tolerance, and if it proliferates more quickly, it induces an immune response. Finally, T cells depend on the integration of at least four signals to effectively differentiate and proliferate: antigen stimulation, co-stimulation, cytokine signaling, and nutrient-sensing.

Second, T cells and other immune cells may both compete with cancer cells for resources and suffer damage or increased mortality from attacking cancer cells (75, 76). This means that T cells may engage in resource competition with their “prey” [known in ecology as intra-guild predation (77, 78)] or engage in a strong form of interference competition, where the antagonism of T cells towards cancer cells entails a fitness cost rather than benefit (79). Furthermore, surviving cancer cells may actually benefit from the death of their neighbors as they can use macro- or micro-pinocytosis to acquire macromolecules following immune-induced death of neighboring cancer cells. As noted above, there is competition for substrate between cancer and immune cells in the resource-limited tumor microenvironment. The cancer cell’s ability to acquire these macromolecules, unlike the immune cells, may give them a significant advantage (80).

Third, the immune system community has diverse cell types that, when included, may generate a “foodweb” that is quite counter-intuitive and far removed from classic predator-prey systems. Initiation of an immune response triggers a cascade of signaling molecules that alter immune cell growth rates before they ever contact the prey. For example, once activated, NK cells respond quickly, inducing target cell death, as well as releasing cytokines and chemokines (81). These directly and indirectly trigger proliferation and recruitment of additional NK cells and other immune cells, including cytotoxic and helper T cells. The decoupling of predator growth rate from prey consumption alters the dynamics of the predator-prey system, which has been exploited clinically. For instance, interleukin-2 (IL-2) is a strong growth signal. It directly binds receptors on immune cells triggering rapid proliferation of the cytotoxic immune cell population. IL-2 therapy has been used to increase the per capita growth rate of T cells *in vivo* and is the key driver of *ex vivo* expansion of tumor infiltrating lymphocytes (TIL) used for TIL-based immunotherapy in cancer (82, 83). In contrast, in nature, predators must directly kill and consume more prey to increase their per capita growth rate.

### Oscillatory Behaviors in Predator-Prey Interactions and the Cycle of Immunoediting

Oscillations are an intrinsic feature of predator-prey interactions (16). Four factors contribute to oscillations in natural predator-prey systems: 1) the functional response (Type II promotes oscillations), 2) time lags between changes in prey population sizes and the numerical responses of the predators (exacerbated when there is a large discrepancy between the generation time of the prey and the generation time of the predator), 3) the magnitude of the indirect feedback between how effective predators are at killing prey and how strong the numerical response is (larger is more destabilizing), and 4) the killing efficiency of the predators (destabilizing). Many of these factors are interrelated. All lead to the key outcomes of the classic one-predator, one-prey species dynamics. The interaction leads to either stable coexistence of prey and predator at positive population sizes, the extinction of the predator and release of the prey population, self-annihilation, where the predators drive the prey extinct and then the predators starve [i.e., Huffakers mites (84), didinium-paramecium (85, 86), or continuous oscillations in the abundance of prey and predator (i.e., spruce budworm epidemics (87), vole cycle (88–90), lynx-hare cycle (91)]. If the immune and cancer cells are in fact in a predator-prey relationship, they should exhibit these properties. However, tumor size oscillations are not typically observed, suggesting that predator-prey model may not be appropriate unless some important considerations are missing.

The regimes predicted by predator-prey models (**Figure 2**) parallel the phases of the immunoediting process (92, 93). Immunoediting has three main phases: 1) elimination, whereby cytotoxic immune cells are highly efficient and eliminate cancer cells; 2) equilibrium whereby the immune system is less efficient and contains but does not eliminate the tumor, and finally 3) an escape phase, where the immune system becomes inefficient and incapable of suppressing cancer growth, leading to tumor growth.

**Figure 3:**
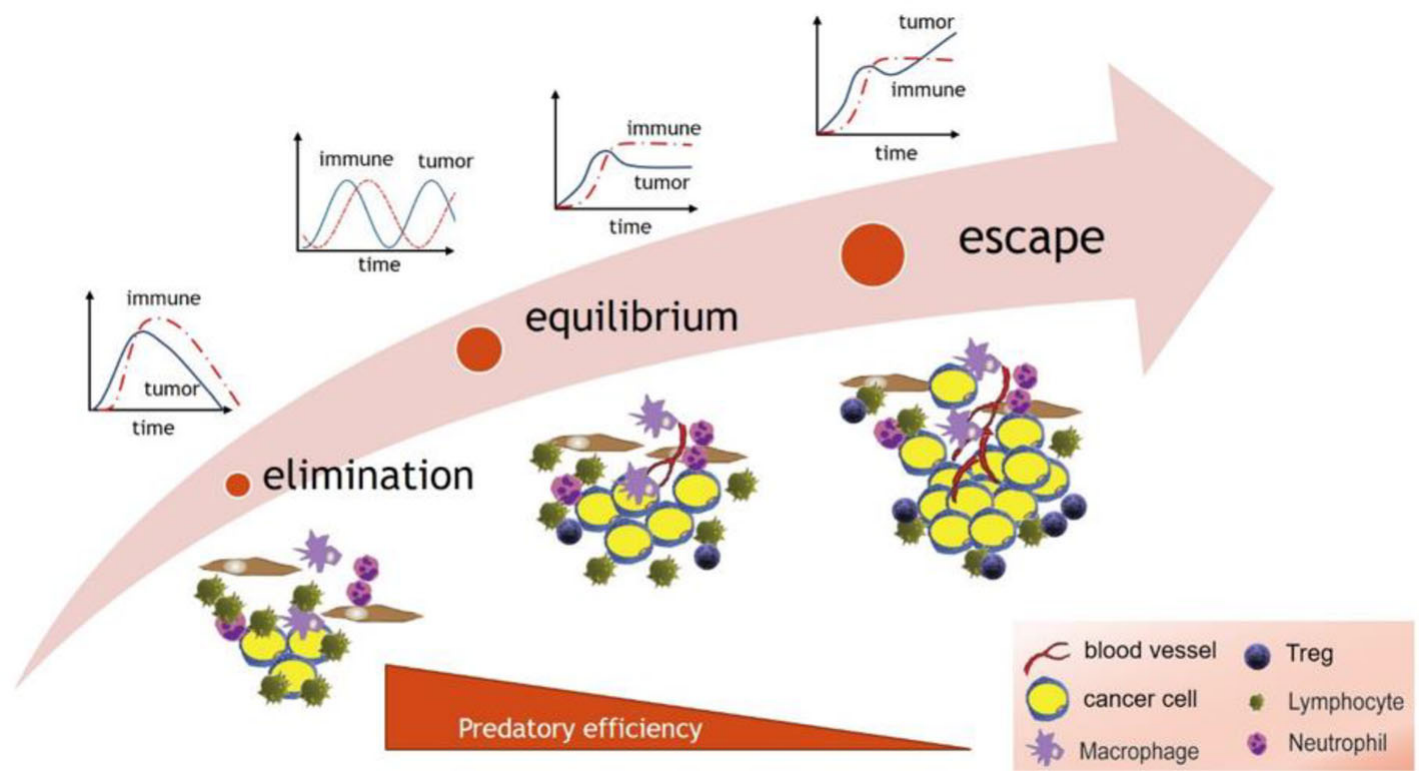
The dynamical regimes of cancer immunoediting paralleled by the sequence of regimes predicted by predator-prey models in response to decreasing predator efficiency.

What is not seen in the cancer immune interaction are stable oscillations on a population wide scale. During immunoediting, it is possible that there exists a transitional oscillatory regime between elimination and equilibrium (equivalent to Region 3 in **Figure 2**), which might be missed due to its occurrence early in disease progression. Or, it is also possible, when examined on a smaller scale, that predator-prey oscillations may occur between subpopulations of antigen specific cytotoxic (CD8) T cell pools and tumor cell clones expressing the corresponding antigens (94); however, they may be missed due to the small amplitude or small spatial scale of resulting oscillations. Therefore, it is possible that transitions between different regimes predicted by the model in **Figure 2** and outlined in **Figure 3** can be explained by diminishing efficiency of the predator as described above. Oscillatory regimes are likely “sandwiched” between elimination and equilibrium and thus should not disqualify predator-prey framework from providing a conceptual framework for describing tumor immune interactions.

Notably, large scale oscillations likely are not seen in tumor-immune interactions because of the threat of autoimmune responses (damage to normal cells), subsidy of immune cells from outside of the tumor, and a weak feedback between the local killing of cancer cells and the recruitment of additional T-cells to the locality. The threat of autoimmunity likely explains why T cells tend to be very inefficient predators of cancer cells. The dynamics of the immune system are not closed, and instead include signaling and recruitment that is host-wide. Migration or recruitment of predators from outside of the area of the prey dynamics will generally be either stabilizing or will result in prey extinction. Finally, the interaction of local and host-wide control of immune responses does not let the local population of T-cells proliferate in direct relation to their killing of cancer cells. This may be a whole-organism adaptation to prevent oscillations in immune and pathogen cell populations.

Maintaining whole-body homeostasis in the face of immune cell – pathogen oscillations may be challenging. Cycling investment into immune cell production may compromise other body functions, and pathogen outbreaks may prove debilitating or lethal. For the whole organism it may be adaptive to keep the pathogen suppressed at a low population size. In fact, it may be to the pathogen’s advantage to have fluctuations as a way of overwhelming the immune system temporally to increase the pathogen’s transmission rate, a hypothesis that remains to be investigated. It is noteworthy that such cycling dynamics can be seen in malaria (95, 96), suggesting that these dynamics may occur in certain diseases. Whatever the dynamical outcomes, the cancer-immune interaction involves not just one cell type of each but a “food-web” or “immune-web” of diverse immune and cancer cell types.

## Ecological Relationships Within the Immune-Web of the Tumor and Immune System

**Figure 4:**
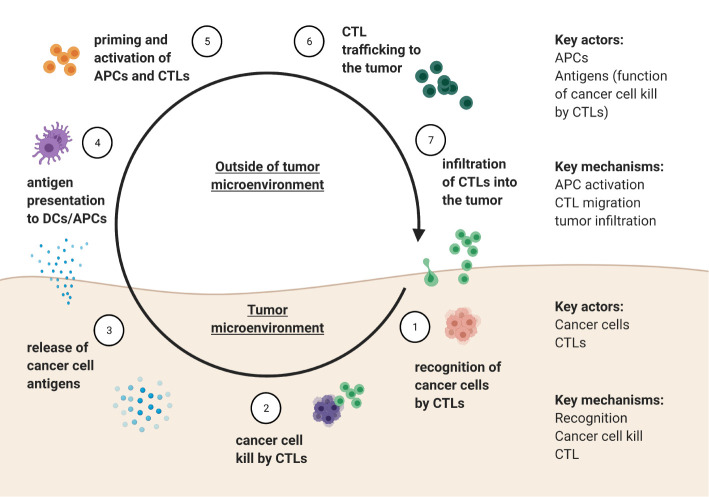
Summary of the tumor-immune cycle adapted from Mellman and Chen (56), delineating key actors and mechanisms that defined the tumor-immunity cycle within and outside of the tumor microenvironment.

The cancer-immunity cycle as described by Mellman and Chen (97) outlines seven key steps involved in tumor-immune interactions: 1) recognition of cancer cells by CTLs, followed by 2) killing of cancer cell by CTLs, resulting in 3) release of cancer cell antigens, which occur in the tumor microenvironment. The antigens need to then be 4) encountered by antigen presenting cells, such as dendritic cells (DCs) outside of the tumor microenvironment, leading to 5) priming and activation of APCs and T cells. Activated CTLs then 6) migrate to and 7) infiltrate the tumor, where the cycle can repeat. A summary of how these processes translate into populations, variables, and dynamics that can then be modeled mathematically is shown in **Figure 4**. The community of different immune cells form an immune-web whereby each cell type can directly or indirectly influence the growth rates of cancer cells and the other immune cell types.

Many of the interactions within the cancer-immunity cycle go beyond strict predator-prey relationships. Interactions between cytotoxic cells (including CD8+T cells, NK cells, macrophages, etc.) and cancer cells constitute just one of several types of ecological relationships that occur within the larger tumor ecosystem. Interactions between populations, when captured in a mathematical framework, include competitive interactions (negative inter-type effects), commensalisms (when one type benefits from the interaction but causes no good or harm to the other), amensalisms (when a type harms the other species at no cost or benefit to itself), or mutualisms (positive inter-type effects usually through the exchange of resources or through the production of public goods). Within the community of immune cell-cancer cell interactions, one can find examples of all of these community modules (predator-prey, competition, commensalism, amensalism, and mutualism).

The specific type of interaction between different cell types is revealed by interaction coefficients (see **Box 1** and **Table 2**). Within a matrix of interaction coefficients, each coefficient describes how changing the population size of one type (represented by the column) influences the population growth rate of another type (represented by the rows). The diagonal coefficients describe intra-type effects, and the off-diagonals describe inter-type effects. As with predator-prey models, the type of interaction will predict different dynamics and stability properties, allowing one to evaluate in a rigorous way the various aspects of cancer-immune ecology. The question of interest should drive the level of abstraction and the number of different immune and cancer cell types to include. The interaction matrices in **Table 2** can provide a touchstone, from which to evaluate the suitability of the model and the reasonableness of its predictions. The interaction matrices also identify when the tumor-immune interaction is predator-prey like or not.

Box 1 **Constructing a matrix of interaction conefficients.**

We can imagine a matrix of interaction coefficients describing the direct effect of each cell type, such as cancer and immune cells (the matrix columns) on the population dynamics of other cells types (the matrix rows). Hence, a direct effect, *a*_ij_, describes the effect of changing the population size of cell type j on the growth rate of cell type i. When i = j then the effects are intra-type (diagonal elements) and when different, the effects are inter-type (off diagonal elements).

Example: $\left(\begin{array}{ll} a_{11} & a_{12}\\ a_{21} & a_{22} \end{array}\right)$

Elements a_11_ and a_22_ refer to intra-species dynamics (within populations of cancer or immune cells), while a_12_ and a_21_ describe inter-precies dynamics (between cancer and immune cells). The sign of the matrix elements can reveal the nature of relationship between the variables (see **Table 2**).

**Table 2:** Interaction matrices and corresponding diagnostics for mathematical models of ecological systems.

Interaction	Interaction matrix		Diagnostics
**Predator-prey**	$\left(\begin{array}{c} a_{11}\\ + \end{array}\;\begin{array}{c} -\\ a_{22} \end{array}\right)$	*a*_11_ = 0 *a*_22_ = 0	Lotka-Volterra predator-prey type model
		*a*_11_ < 0 *a*_22_ = 0	Type I functional response or inefficient predator with Type II response
		*a*_11_ > 0 *a*_22_ = 0	Occurs if safety in numbers is greater than intraspecies competition
		*a*_22_ < 0	Self-regulatory predator or ecology of fear
**Competition**	$\left(\begin{array}{ll} a_{11} & -\\ + & a_{22} \end{array}\right)$	*a*_11_, *a*_22_ likely negative	If interaction amplifies *a*_12_ and diminishes *a*_21_, there is intraguild predation
			If interaction amplifies both *a*_12_ and *a*_21_, there is strong interference competition
		*a*_11_ > 0 *a*_22_ < 0	Allee effect: transitional behavior, where first species either goes to extinction, or grows past a survival threshold
**Commensalism**	$\left(\begin{array}{ll} a_{11} & -\\ - & a_{22} \end{array}\right)$	*a*_11_ < 0 *a*_22_ ≤ 0	Interaction causes second species to benefit from the interaction but causes no good or harm
**Amensalism**	$\left(\begin{array}{c} a_{11}\\ - \end{array}\;\begin{array}{c} 0\\ a_{22} \end{array}\right)$	*a*_11_, *a*_22_ can be either positive, negative or zero	Interaction causes first species to harm second species at no cost or benefit to self
**Mutualism**	$\left(\begin{array}{ll} a_{11} & -\\ + & a_{22} \end{array}\right)$	*a*_11_ ≤ 0 *a*_22_ ≤ 0	The interaction is mutually beneficial to both species

### When Tumor-Immune Interactions Do and Do Not Fall Within the Predator-Prey Framework

If the goal of the research question is to focus on the tumor-immune community module within the tumor microenvironment, then we propose the following as the minimally sufficient model of tumor-immune interactions:

Primary variables: cancer cells, activated effector (cytotoxic) cells, antigen presenting cells (APCs).

Mechanisms of interactions of primary cell types:

1. Cancer cells grow up to some carrying capacity (which may become dynamic if needed).
2. Cytotoxic (effector) cells can kill cancer cells.
3. Killing cancer cells by cytotoxic cells can stimulate APCs.
4. Population of effector cells increases through interactions with APCs.
5. Population of effector cells decreases in the absence of cancer cells.
6. Effector cells can become tolerized or exhausted as a result of killing cancer cells.

**Figure 5:**
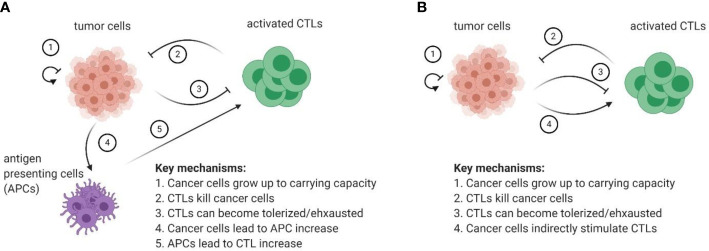
**(A)** Proposed set of minimally sufficient variables and mechanisms to be included in future models of tumor-immune interactions. **(B)** Ecological abstraction of the full model, where it is assumed that interactions with APCs and subsequent T cell activation reach a quasi-steady state before impacting interactions between CTLs and cancer cells in the tumor microenvironment. The resulting model has features of a classical predator-prey type model with a caveat that CTLs are indirectly stimulated by cancer cells.

**Figure 5A** provides a schematic of such a model.

Note that based on community modules (**Table 2**), the interaction between cancer cells and cytotoxic cells is one of interference competition in addition to possible competition for resources. APCs act as a commensal for cytotoxic cells, and cancer cells as a commensal for APCs. This three cell-type community can see the coexistence of all three populations, or the elimination of cancer cells, or the elimination of cytotoxic cells. Furthermore, there can exist alternate stable states, where either the cancer cells exclude the cytotoxic cells or vice-versa, depending on which cell type starts out as relatively more frequent. If all three types can persist together, then the population dynamics may converge to a stable equilibrium or show permanent oscillations. The outcome depends upon the strength of self-regulatory feedbacks of cytotoxic cells and APCs on themselves, and upon the strengths of the inter-cell-type interaction coefficients. Interestingly, when the cytotoxic cells and cancer cells exhibit an extreme form of interference competition, then their community module suppresses oscillatory population dynamics, and leaves open the possibility of alternate stable states.

It is tempting to see cytokines and cell signaling molecules as central to any model of immune-cancer dynamics (26). While these may be essential components of the system, we do not necessarily see the need to model their dynamics as separate populations of molecules. It is possible in this case to take advantage of time scale separation. In this way, the cytokine dynamics can be rolled into the equations for the three cell types. For instance, if cytokine signaling occurs on a scale of minutes but cell growth occurs on a scale of days, then relative to cell dynamics, cytokine signaling achieves a quasi-steady state for any population of the three cell types. If this cytokine steady state can be approximated, then the level of cytokines and their effect on parameters can be directly included in the three-cell-type model of population dynamics without having to explicitly consider a fourth dynamical equation.

Furthermore, the immune-cancer interaction can still be modelled using just two populations: one for cytotoxic and one for cancer cells. For instance, if the question of interest focuses on the dynamics in the tumor microenvironment, it can be assumed that the interaction with antigen-presenting cells and subsequent T cell activation reaches a quasi-steady state before it can affect the cancer cell dynamics that occur in the tumor microenvironment. If so, the three-dimensional model described in **Figure 5A** reduces to a predator-prey like model, where CTLs are indirectly stimulated by cancer cells (i.e., there is still no direct conversion of prey biomass into predator). The diagram of this abstracted cancer cell – immune cell interaction can be found in **Figure 5B**.

This kind of ecological abstraction can reduce the number of equations to two, and the cancer cell-immune cell interactions may well fit a community module that is predator-prey. However, it is important to note that this technique still implicitly includes the intermediate immune activation step, and thus the resulting predator-prey-like system does not violate the caveat of immune cells “not working on commission”.

Whether the resulting two cell-type system conforms to competition or predator-prey type models depends on the magnitude of the positive indirect effect of cancer cells on cytotoxic cells (cells stimulate APCs that stimulate cytotoxic cells) relative to the negative direct effect of cancer cells on cytotoxic cells *via* resource competition or immune cell exhaustion. If the indirect effect is smaller than the direct one, then the system will be one of competition. Such a two-species system will not see oscillatory population dynamics, and outcomes will generally include coexistence, elimination of one type by the other independent of initial populations, or alternate stable states, where the outcome depends on the starting population sizes. If the indirect effect is larger than the direct, then the interaction between the cancer cell population and cytotoxic immune cells will be predator-prey with all of the concomitant consequences.

The community of cell types may grow further with the inclusion of two or more cancer cell types with different susceptibilities to one or more types of cytotoxic cells. In this way, the model may expand to two-prey and one predator, two prey and two predators, etc., thus expanding the number and types of community modules describing the immune-web. Model expansions should align with the research question. The two prey and one predator type model leads to *apparent competition* between the two cancer cell types (98, 99), and the two prey-two predator system can lead to *indirect mutualism* between the two types of cytotoxic cells if they specialize on different cancer cell types (100).

## Discussion

Primary and secondary resistance to immunotherapy is a significant clinical problem. Theoretical models are needed to define the underlying evolutionary dynamics of treatment resistance to optimize outcomes. Various mathematical models have been created to capture different aspects of tumor-immune ecology. These include introducing dynamic carrying capacities for tumor and immune cells (101), introducing treatment, IL-2 and IL-12 cytokines (102), incorporating ecological abstraction in APC maturation (27), incorporating NK cells, Tregs and IL-2 cytokines (103), as well as larger scale models that include various stages of activation of effector cells, helper cells, mature Tregs, and various cytokines, such as TGF-beta and IL-10 (26), among others. Various ecological interactions can predict different types of dynamics, which may not be easily identifiable otherwise (i.e., oscillatory regime as part of the sequence of immunoediting in predator-prey type models). As a field, we can take advantage of the rich tradition of eco-evolutionary modeling from ecology to understand cancer-immune interactions and to optimize treatment.

It is important to note, however, that mathematical models are most useful when they are tailored to answer specific questions, just as experiments are designed to test specific hypotheses. The question should therefore drive design of the model and the number of populations and associated interactions. Tailor the model to the question and not the other way around. However, while being tailored for specific questions, these models need to be based on a unified framework, such as the one proposed here, to enable building on the existing body of knowledge rather than starting every time anew.

What is the relevance of the models presented here to the non-math oncology community? Immunology is a complex and humbling field of study. Cancer immunologists and oncologists deal with evolving cancer cells, growing or shrinking tumors, and a changing immune system. This is compounded by the emergence of immunotherapy resistance and use of immunotherapies in combination with other treatment modalities. Additionally, immunology is not a stagnant field. The concepts that define ‘self’ and ‘non self’ and determine what can elicit an immune response are shifting. In the last 30 years we have seen the introduction of the danger model, the discontinuity theory of immunity, the growth-threshold conjecture, and a call to redefine the immunological self (70, 71, 73, 104–106).

Theoretical modeling has an increasing role to play in meeting the challenges of modern cancer immunology. For instance, examining the classic predator-prey system in the context of cancer immunology and contrasting it with the ecology models has revealed the obvious importance of predator efficiency in eliminating the prey in a structured and (surprisingly simple) mechanistic way. Increasing immune cell efficiency against tumor cells is the primary goal of current immunotherapies including tumor vaccines, checkpoint inhibition, and genetically engineered immune cells. By comparing the divergent roles of biomass conversion in the two systems, we can show the importance of resource competition between predators (immune cells) and prey (tumor cells). Thus, it may be necessary to alter the TME to increase immunotherapy efficacy (107). Additionally, the lack of reliance on prey consumption for predator growth in cancer-immune models highlights the importance of the “immune-web” created by other members of the immune system and associated cytokines. While direct alterations of this web, such as *via* systemic IL-2, have not proven effective, more nuanced or localized targeting of the immune-web may serve to support other immunotherapies, hypotheses that could be evaluated using complementary experimental and modeling approaches.

Modeling also allows one to compare predictions against observations, thereby making us rigorously question our understanding of the underlying biology. For instance, the less explored dynamics, oscillations in predator and prey population levels, call into question why we do not see the same oscillations in immune cell and cancer cell populations that are intrinsic to the ecology models. Here we provide a few hypotheses, but more work is needed. It may not be immediately obvious why an immunologist would want to answer this question. However, the immune response is a numbers game. Current dogma states that increased antigen levels directly lead to increased immune activation, and that antigen removal equals diminishing immune activation. If these dynamics were the only ones, then we would see oscillations. If the host response is designed to avoid oscillations to regulate co-evolution of pathogens or avoid immunopathology the cancer cells are likely utilizing these processes to circumvent the anti-tumor immune response. Since we seek to reverse the immunoediting process and promote the elimination phase, we must understand the interim phases and their contribution to immunotherapy resistance, and modeling can help.

## Data Availability Statement

The original contributions presented in the study are included in the article/supplementary material. Further inquiries can be directed to the corresponding author.

## Author Contributions

IK, KL, and JB contributed to problem formulation and study design. All authors contributed to the article and approved the submitted version.

## Funding

This research was partially supported by grants U54 CA143970, R01 CA077575, and U01CA232382 (to RG and JB).

## Conflict of Interest

IK is an employee of EMD Serono, US subsidiary of Merck KGaA. Views presented in this manuscript do not necessarily reflect the views of EMD Serono.

The remaining authors declare that the research was conducted in the absence of any commercial or financial relationships that could be construed as a potential conflict of interest.

## Publisher’s Note

All claims expressed in this article are solely those of the authors and do not necessarily represent those of their affiliated organizations, or those of the publisher, the editors and the reviewers. Any product that may be evaluated in this article, or claim that may be made by its manufacturer, is not guaranteed or endorsed by the publisher.
